# Comparison of Aroma and Taste Profiles of Kiwi Wine Fermented with/without Peel by Combining Intelligent Sensory, Gas Chromatography-Mass Spectrometry, and Proton Nuclear Magnetic Resonance

**DOI:** 10.3390/foods13111729

**Published:** 2024-05-31

**Authors:** Bingde Zhou, Xiaochen Liu, Qiuyu Lan, Fang Wan, Zhibo Yang, Xin Nie, Zijian Cai, Bin Hu, Junni Tang, Chenglin Zhu, Luca Laghi

**Affiliations:** 1 College of Food Science and Technology, Southwest Minzu University, Chengdu 610041, China; zhoubingde123@outlook.com (B.Z.); lxc050922@outlook.com (X.L.); lanqiuyu211@gmail.com (Q.L.); wfwfwfo1@outlook.com (F.W.); yangzhibo@stu.sicau.edu.cn (Z.Y.); caizijian@swun.edu.cn (Z.C.); junneytang@swun.edu.cn (J.T.); 2 Department of Agricultural and Food Sciences, University of Bologna, 47521 Cesena, Italy; 3 College of Food, Sichuan Agricultural University, Ya’an 625014, China; hubin2555@sina.com; 4 College of Food Science and Technology, Sichuan Tourism University, Chengdu 610041, China; niexin@sctu.edu.cn

**Keywords:** kiwi wine, kiwifruit peel, fermentation, flavoromics, metabolomics

## Abstract

Kiwi wine (KW) is tipically made by fermenting juice from peeled kiwifruit, resulting in the disposal of peel and pomace as by-products. However, the peel contains various beneficial compounds, like phenols and flavonoids. Since the peel is edible and rich in these compounds, incorporating it into the fermentation process of KW presents a potential solution to minimize by-product waste. This study compared the aroma and taste profiles of KW from peeled (PKW) and unpeeled (UKW) kiwifruits by combining intelligent sensory technology, GC-MS, and ^1^H-NMR. Focusing on aroma profiles, 75 volatile organic compounds (VOCs) were identified in KW fermented with peel, and 73 VOCs in KW without peel, with 62 VOCs common to both. Among these compounds, rose oxide, D-citronellol, and bornylene were more abundant in UKW, while hexyl acetate, isoamyl acetate, and 2,4,5-trichlorobenzene were significantly higher in PKW. For taste profiles, E-tongue analysis revealed differences in the taste profiles of KW from the two sources. A total of 74 molecules were characterized using ^1^H-NMR. UKW exhibited significantly higher levels of tartrate, galactarate, N-acetylserotonin, 4-hydroxy-3-methoxymandelate, fumarate, and N-acetylglycine, along with a significantly lower level of oxypurinol compared to PKW. This study seeks to develop the theoretical understanding of the fermentation of kiwifruit with peel in sight of the utilization of the whole fruit for KW production, to increase the economic value of kiwifruit production.

## 1. Introduction

Kiwi wine (KW) is a fermented beverage derived from kiwifruit that has a low alcohol content, making it an attractive option for consumers seeking low-alcohol beverages [1]. Its increasing popularity has led to a growing number of publications on its production, which have determined that the quality of KW is influenced by various factors, including the fermentation strains, raw materials, and pre-treatment methods employed [2,3,4]. However, most of these studies focused on KW fermented from peeled and pressed kiwifruits. It is worth noting that the peel and pomace, considered by-products in KW production, account for 20–40% of the whole fruit’s weight [5,6]. Consequently, the large volume of peel and pomace not only increases disposal costs but also exacerbates waste management challenges for the KW industry.

To optimize resource usage, researchers are increasingly experimenting the production of fruit wines by fermenting pomace from a variety of fruits. For instance, the substitution of 75% grape pomace with black carrots during the fermentation of shalgam juices enhances the final product’s concentrations of resveratrol, catechin, rutin, kaempferol-3-O-glucoside, isorhamnetin-3-O-glucoside, cyanidin-3-O-glucoside, petunidin-3-O-glucoside, and malvidin-3-O-glucoside [7]. Lindsay et al. investigated the fermentation of the pomace of apple, orange, and carrot using *Brettanomyces bruxellensis*. The results showed that over 400 volatile metabolites were transferred into the final product or generated during the fermentation process. Additionally, carrot pomace was identified as a natural source of phenylalanine, which can lead to phenyl ethanol, with a pleasant rosy scent [8]. The pomace of kiwifruit contains phenols, organic acids such as quinine and caffeic acid and their derivatives, antioxidant components, and various aroma precursors [9,10]. The kiwifruit peel contains beneficial components, including chlorophyll, polyphenols, and flavonoids, which contribute to its antioxidant activity [7]. Furthermore, it is worth highlighting that the peel of the kiwifruit contains a higher concentration of phenols, flavonoids, and ascorbic acid compared to the pulp [8]. Based on the kiwifruit peel’s edible and nutritive properties, the application of kiwifruit peel in the fermentation of KW is appealing, potentially reducing the production of wastes and increasing the nutritional value of KW. Liu et al. fermented whole kiwi fruits with *Cyclocarya paliurus*. The resulting *Cyclocarya paliurus*–kiwi composite fruit wine exhibited significantly higher O_2_^−^ scavenging activity, total flavonoid content (TFC), and total phenolic content (TPC) compared to the KW obtained only from the pulp and to two commercial KWs [11]. Nevertheless, there has been a paucity of research work conducted on the utilization of kiwifruit peel and pomace for the fermentation of KW, particularly in terms of flavor profile.

The flavor of fruit wines, mainly determined by aroma and taste characteristics, is one of the most important indicators of their quality. The principal volatile organic compounds (VOCs) in KW, such as esters, alcohols, and acids, are considered the main components of the delightful aroma of wine [12]. Using gas chromatography-mass spectrometry (GC-MS), Lan et al. analyzed the aroma characteristics of commercially available KW [13], identifying 50 key VOCs, with the most concentrated being ethyl caprylate. Sun et al. employed GC-MS to characterize VOCs in KW. Their findings indicated that adding a yeast blend during fermentation increased esters and aldehydes, as well as floral and sweet fruity flavors of KW [14].

In addition to volatile compounds, many non-volatile classes of molecules, such as organic acids, amino acids, and sugars, significantly shape the taste profile of KW. However, these compounds have a variety of functional groups, making their simultaneous and rapid analysis challenging with a single analytical platform. Proton nuclear magnetic resonance (^1^H-NMR) is highly advantageous in this context because its operational principles inherently allow for quantitative analysis regardless of the chemical characteristics of the observed molecules. For example, Zhang et al. successfully employed ^1^H-NMR to investigate the impact of *Saccharomyces cerevisiae* strains on the sensory and flavor characteristics of KW.

To the best of our knowledge, there is a lack of research on the impact of kiwifruit peel on the aroma and taste profiles of KW. This work may contribute filling this knowledge gap by comparing the flavor characteristics of KW obtained from peeled and unpeeled kiwifruits. For this purpose, E-tongue, GC-MS, and ^1^H-NMR were employed. This study could set the basis for the theoretical understanding of the fermentation of kiwifruit with peel, facilitating the utilization of the whole fruit for KW production and increasing the economic value of kiwifruit production.

## 2. Materials and Methods

### 2.1. KW Production

KW was produced by adapting the method described by Zhang et al. to the present experimental plan [7]. Uniformly sized and intact Pujiang (Chengdu, China) kiwifruits were selected and cleaned. To obtain kiwi wine from unpeeled fruits (UKW), 100 g of kiwifruits was pressed, mixed with 100 mL of de-ionized water, and fermented into KW. To obtain kiwi wine from peeled fruits (PKW), 100 g of pomace was pressed, mixed with 100 mL of de-ionized water, and fermented into KW. Prior to UKW and PKW fermentation, 60 mg/L metabisulphite (Shanghai yuanye Bio-Technology Co., Ltd., Shanghai, China) and 40 mg/L pectinase (Shandong Lonct Enzymes Co., Ltd., Linyi, China) were added for color protection and enzymatic digestion (12 h). Subsequently, sugar (Xinwangbao Food Co., Ltd., Chengdu, China) was added to reach 22 °Brix. After the inoculation of yeast (0.4 g/L, *Saccharomyces cerevisiae* RW, Angel Yeast Co., Ltd., Yichang, China), fermentation was performed at 25 °C for 30 days. The two productions were repeated five times each.

### 2.2. Analysis of Aroma Profile in KW

An analysis of aroma was performed according to Zhu et al., with minor modifications [15]. A 20 mL headspace injection vial was used to pour 5 mL of KW. A 50/30 µm DVB/CAR/PDMS extraction header was used to enrich the flavor compounds under extraction adsorption conditions at 50 °C for 30 min. The enriched KW was then subjected to GC-MS analysis.

The chromatographic column used was a TG-WAXMSB (30 m × 0.25 mm, 0.25 µm). The inlet temperature was set to 230 °C in non-split mode. The column temperature was held at 40 °C for the initial 3 min. It was then ramped up to 180 °C at a rate of 6 °C/min and held for 2 min. The temperature was then increased to 230 °C at 10 °C/min and held for 6 min. The carrier gas used was helium (purity > 99.999%) with a flow rate of 1.0 mL/min. The electron ionization source was set to an electron energy of 70 eV, with an ion source temperature of 250 °C and a transmission line temperature of 230 °C. The mass scan range was set to 30–550 *m*/*z* with a scan time of 2 s, using full scan mode in the EI ion source. The retention indices of these compounds relative to standard alkanes (C8–C26) were calculated by comparing the experimental mass spectra with the NIST 11 mass spectral library. Subsequently, the results were compared with those in the database, and compounds were identified only if they had a match of more than 90%.

### 2.3. Analysis of Taste Profile in KW

#### 2.3.1. E-Tongue Analysis

E-tongue analysis was performed according to Zhang et al., with slight modifications [16]. A specific E-tongue beaker was used to hold 80 mL of KW. The E-tongue determination conditions were then set, with a signal acquisition time of 120 s, a stirring rate of 60 r/min, and an analysis process of 3 min. At the end of each analysis, the sensors were cleaned with deionized water for 30 s to remove any residue that could affect the subsequent determinations, thus ensuring accuracy and reliability. To further improve accuracy and reduce errors, each sample was tested five times. The data from the last three stable tests were analyzed.

#### 2.3.2. ^1^H-NMR Analysis

The method of Zhu et al. was used as a basis for ^1^H-NMR analysis, with some adaptations [15]. A 0.5 mL sample of KW was centrifuged (18,630× *g*) at 4 °C for 15 min. The obtained supernatant (0.35 mL) was mixed with distilled water (0.35 mL) and NMR analysis solution (200 μL). The mixture was then centrifuged under the same conditions. The ^1^H-NMR determination was performed using a 600.13 MHz AVANCE III HD NMR spectrometer. The carrier frequency was set to 600.13 MHz, and the detection temperature was set to 298 K. The spectral width was set to 10, covering the proton signals from all the organic compounds in the samples, comprehensively reflecting the chemical structure information of the samples. The recycle delay was set to 5 s, to ensure an adequate recovery time between each scan. Data acquisition comprised 32,000 data points. To suppress interferences from the water’s signal, a CPMG sequence with presaturation was employed. The number of scans was set to 128.

The NMR profiles were processed using Topspin 3.5 with Chenomx 8.4 software. Baselines were manually adjusted in Topspin, and spectra were converted to ASCII files and then exported to the in-house R programming language for further processing. This included chemical shift calibration and the removal of the water’s residual signal. Molecule identification was conducted by comparing peaks’ chemical shifts, multiplicity, and shape with the Chenomx standard peaks library. Quantitative analysis was performed by rectangular integration.

### 2.4. Statistical Analysis

Statistical analysis was performed in the R computational language. Prior to the univariate analyses, the distribution of the data was brought to normality according to Box and Cox [17]. T-tests were employed to identify significant differences between groups, with a significance level of *p* < 0.05. To obtain an overall view of the data, robust principal component analysis (rPCA) models were set up based on the concentrations of molecules. For each rPCA model, a score plot and a Pearson’s correlation plot of the loadings were calculated to highlight the structure of the data and find out the relationships between variables and the model’s components.

Pearson correlations among ^1^H-NMR data were looked for by taking advantage of the corr.test function in R. Relationships between ^1^H-NMR and sensory data were calculated through a Mantel’s test, taking advantage of the online tool www.omicstudio.cn (accessed on 13 April 2024).

## 3. Results

### 3.1. Analysis of Aroma Profile in KW

To investigate the aroma profiles of both UKW and PKW samples, VOCs were analyzed by GC-MS. We characterized a total of 86 molecules, including esters (31), aldehydes (8), alcohols (14), acids (7), ketones (4), and others (22), as listed in Table S1.

**Figure 1:**
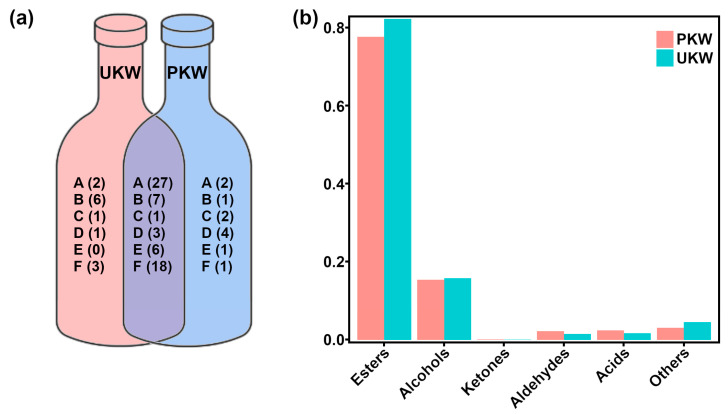
Veen plot (**a**) of the number of species of VOCs in UKW and PKW. The letters A, B, C, D, E, and F represent the classes of VOCs, namely esters, alcohols, ketones, aldehydes, acids, and others, respectively. Bar plot (**b**) of the contents of VOCs in UKW and PKW.

In PKW and UKW samples, we identified, respectively, 73 and 75 VOCs, with 62 shared VOCs, as shown in Figure 1a. Of the 27 esters shared, ethyl caprylate and ethyl caproate were the most abundant, therefore mostly contributing to the aroma of KW. Figure 1a shows that 13 VOCs were unique for UKW samples, while 11 VOCs were unique to PKW. In detail, ethyl 2-hydroxy-4-methylpentanoate and ethyl 9-decenoate were detected only in PKW, whereas ethyl (E)-4-decenoate and propanoic acid, 2-methyl-, 1-(1,1-dimethylethyl)-2-methyl-1,3-propanediyl ester was detected only in UKW. For the alcohols, 1-pentalnol was found only in PKW and was the most abundant of this class of compounds. Interestingly, 1-decanol, 3-methyl-1-butanol, 5-methyl-3-heptanol, 1-octen-3-ol, linalool, and trans-2-hexenol could not be detected in PKW. Focusing on the aldehydes, 2,5-dimethylbenzaldehyde was detected only in UKW samples, while undecanal, octanal, 2-undecenal, and hexanal were found only in UKW samples. Similarly, lauric acid was detected only in UKW samples.

Figure 1b illustrates the relative presence of volatile categories in UKW and PKW. Esters were the major VOCs in both UKW and PKW, representing more than 75% of all the molecules characterized. UKW was characterized by a higher relative abundance of esters, alcohols, and others, along with a lower relative abundance of ketones, aldehydes, and acids. To further investigate the dissimilarities in VOCs between UKW and PKW, *t*-tests were conducted on 62 VOCs that were shared by UKW and PKW samples. As illustrated in Table 1, 7 VOCs showed significantly different concentrations (*p* < 0.05). Among them, ethyl heptanoate, isobutyl acetate, hexyl acetate, and rose oxide exhibit relative odor activity values (ROAVs) higher than 1, thus contributing greatly to the aroma profile of KW, as illustrated in Table S2.

**Figure 2:**
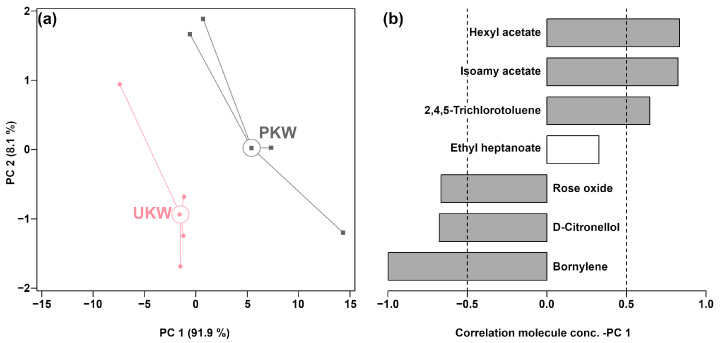
Score plot (**a**) and Pearson correlation plot (**b**) of the loadings of an rPCA model based on molecule concentrations for which significant differences were found by GC-MS.

To obtain an overall visual summary of the differences between the KWs made with and without peel, an unsupervised rPCA model was calculated for the concentrations of the seven VOCs of Table 1, as illustrated in Figure 2.

As shown in Figure 2a, PC 1 accounted for 91.9% of the variance and effectively captured the overall information of the samples, with UKW and PKW samples appearing at negative and positive PC 1 scores, respectively. As shown in the loadings plot (Figure 2b), in comparison to PKW, UKW exhibited elevated concentrations of rose oxide, D-citronellol, and bornylene. Conversely, the contents of hexyl acetate, isoamyl acetate, and 2,4,5-trichlorotoluene were observed to be lower in UKW.

### 3.2. Analysis of Taste Profiles in KW

#### 3.2.1. E-Tongue Analysis

**Figure 3:**
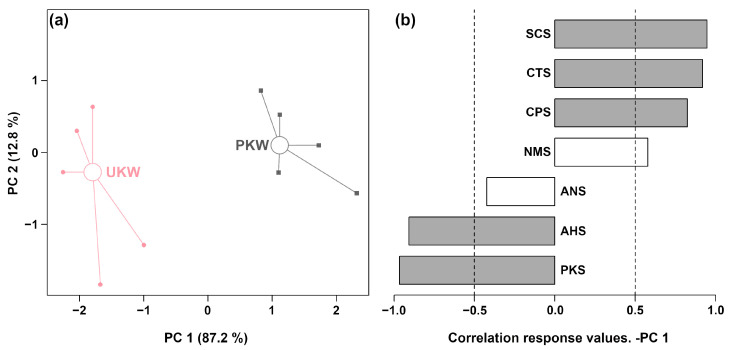
Score plot (**a**) and Pearson correlation plot (**b**) of the loadings of an rPCA model based on E-tongue response data. The sensors are AHS (sourness), CTS (saltiness), NMS (umami), ANS (sweetness), SCS (bitterness), PKS and CPS (reference electrodes).

To characterize the overall taste profile of KW fermented with and without peel, an rPCA model was created using response value data from the E-tongue sensors, as presented in Figure 3.

In Figure 3a, the first principal component accounted for 87.2% of the sample set’s variance, effectively summarizing the overall features of the samples. Notably, samples from PKW and UKW exhibited clear positional peculiarities in PC 1. The results showed that AHS and PKS had significantly higher response values in UKW, while SCS, CTS, and CPS had significantly higher response values in PKW, as shown in Figure 3b.

#### 3.2.2. ^1^H-NMR Analysis

**Figure 4:**
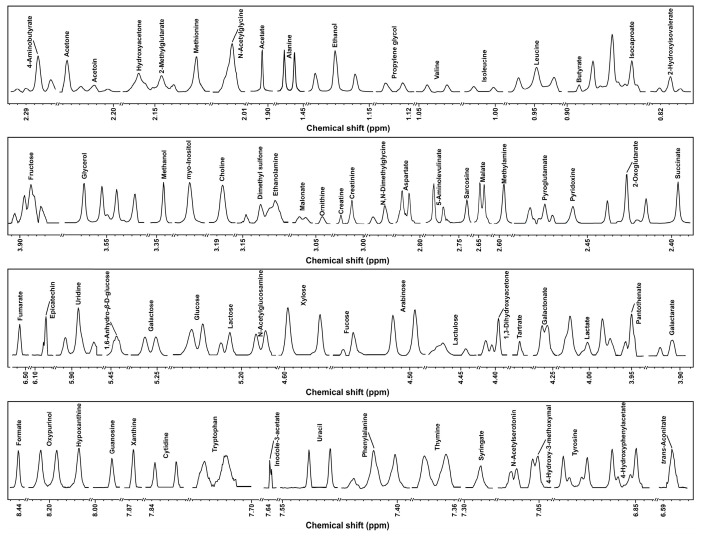
A representative ^1^H-NMR spectrum of KW.

Taking advantage of ^1^H-NMR, a total of 74 molecules were identified and quantified in KW samples. In order to visualize them, representative spectra were plotted for each characterized molecule, as depicted in Figure 4.

**Figure 5:**
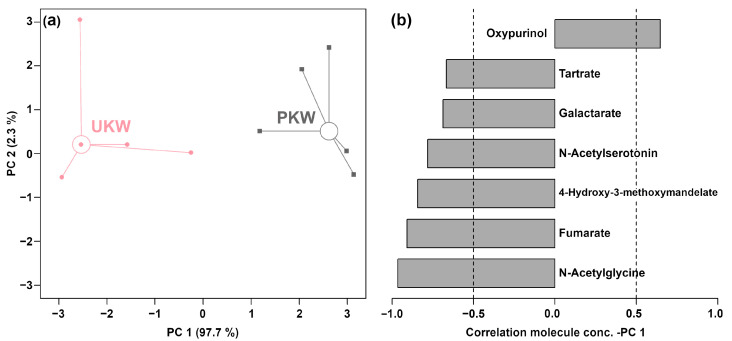
Score plot (**a**) and Pearson correlation plot (**b**) of the loadings of an rPCA model based on molecule concentrations measured by ^1^H-NMR.

Moreover, 74 molecules, including amino acids, peptides and analogs (14), carbohydrates and derivatives (9), organic acids and derivatives (26), nucleosides, nucleotides and analogs (5), alcohols (6), and others (14) were characterized by ^1^H-NMR in the two types of KW, as listed in Table S3. To effectively illustrate the differences in the non-volatile profiles of KW fermented with/without peel, an rPCA model was set up using the molecular concentration determined by ^1^H-NMR, as displayed in Figure 5. Among the quantified molecules, seven were found to be significantly different (*p* < 0.05) between UKW and PKW, namely oxypurinol, fumarate, tartrate, N-acetylserotonin, 4-hydroxy-3-methoxymandelate, N-acetylglycine, and galactarate (Table 2).

As shown in Figure 5a, PC 1 accounted for 97.7% of the variance captured by PC 1. A clear separation between UKW and PKW samples was observed in PC 1. In comparison to PKW (Figure 5b), UKW presented a lower concentration of oxypurinol but higher levels of tartrate, galactarate, N-acetylserotonin, 4-hydroxy-3-methoxymandelate, fumarate, and N-acetylglycine.

#### 3.2.3. Correlation Analysis of E-Tongue with ^1^H-NMR

**Figure 6:**
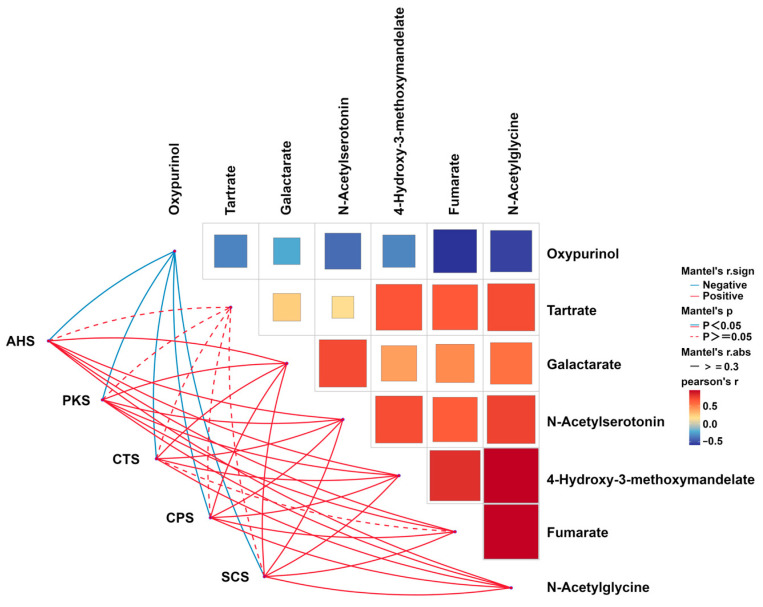
Correlation analysis conducted between the sensors of the E-tongue and molecules quantified by ^1^H-NMR showing differences between the two wines.

To explore the relationship between taste perception and specific KW metabolites, a Mantel’s correlation analysis was conducted between the response of E-tongue’s sensors and molecules detected by ^1^H-NMR, showing a different concentration between the two sets of samples, as shown in Figure 6.

A positive correlation was found among galactarate, N-acetylserotonin, 4-hydroxy-3-methoxymandelate, fumarate, and N-acetylglycine. Among these molecules, galactarate, N-acetylserotonin, 4-hydroxy-3-methoxymandelate, and N-acetylglycine also showed significantly positive correlations with all five sensors of the electronic tongue, while fumarate had positive correlations with all the electronic sensors except CTS. Moreover, there was a significantly negative correlation between fumarate and N-acetylglycine with oxypurinol. All five sensors of the electronic tongue were significantly negatively correlated with oxypurinol.

## 4. Discussion

KW is a processed kiwifruit product that is gaining popularity as it meets consumer demand for low-alcohol beverages [18]. However, KW is typically fermented after the kiwifruit has been peeled and juiced, resulting in a lot of waste from the kiwifruit-processing industry [19]. The use of fruit by-products is a globally popular strategy for promoting sustainable food production [20]. Fruit by-products can be utilized not only as fertilizers but also as raw materials for the recovery of numerous antioxidants or high-value compounds with biological properties [21]. Furthermore, fruit by-products can be utilized in the production of fruit wines [22]. This study attempted to incorporate kiwifruit peel and pomace into the fermentation of KW. The kiwifruit peel contains higher levels of phenols, flavonoids, and vitamin C than the pulp [23], leading to a greater antioxidant capacity and contributing to the improvement of the quality of KW. In addition, the maceration of kiwifruit peel produces more C6 aldehydes, which act as precursors of 1-hexanol in KW, with key flavoring properties [24]. Kiwifruit peel also contains several terpenes and aroma precursors [25]. Consequently, this study concentrated on the impact of the kiwifruit pericarp on aroma and taste and sought to identify a novel approach to the comprehensive utilization of kiwifruit-processing waste.

The main volatile compounds identified in the present study pertained to classes of esters, alcohols, and acids, aligning with previous findings [13]. The ester content of fruit wines primarily results from the fermentation process, with a smaller proportion forming during aging. During fermentation, esters are mainly produced by the condensation of the hydroxyl groups of phenols or aliphatic alcohols with the carboxyl groups of organic acids, catalyzed by yeasts and bacteria. This process adds rich aromas and flavors to fruit wines [26]. Consistent with earlier research, ethyl caprylate and ethyl caproate were the most concentrated esters in KW, imparting fruity and floral aroma [2,13]. Specifically, ethyl caprylate provides fruit wines with a fruity and sweet flavor, with subtle notes of apricot, banana, and pear [13]. Ethyl caproate, a by-product of yeast synthesizing long-chain fatty acids during fermentation, contributes scents of apple, raw banana, pineapple, and floral notes to fruit wines [27]. The content of hexyl acetate was significantly higher in PKW compared to UKW, in agreement with Gao et al., who studied kiwifruit mash fermented with peel [28]. Isoamyl acetate is a key flavor component in yeast-fermented alcoholic beverages [16]. The high concentration of isoamyl acetate in PKW resulted in a more pronounced banana flavor than in UKW [29]. Ethyl 2-hydroxy-4-methyl valerate indirectly effects fruit wine aromas by enhancing the fruity character of blackberry and fresh fruit aromas through synergistic interactions with other substances [30].

Aldehydes produced during fruit wine preparation and fermentation through oxidation, play a crucial role in the flavor of fruit wines. They have a low odor threshold, and most have a distinct fatty aroma [31]. Among them, 2,5-dimethylbenzaldehyde is unique to UKW and gives it a nutty, almond flavor [32]. A distinctive feature of PKW is the presence of undecanal, octanal, and hexanal, which impart a cereal, citrus, and grassy aroma to the product [33,34]. Additionally, 2-undecenal in PKW is often associated with the oxidation of fruit wines. However, this does not affect its quality at low concentrations, while adding an orange peel aroma [35].

Alcohols are a class of VOCs indispensable in fruit wines, not only because they give a pleasant aroma per se but because they contribute to the effectiveness of other aromas’ components [36]. During the production of fruit wines, yeast produces higher alcohols via catabolic and anabolic pathways. At the same time, small amounts of higher alcohols are also produced by reducing the corresponding aldehydes [37]. UKW had a greater variety of higher alcohols than PKW, as revealed by GC-MS. In particular, compared to UKW, PKW contained high levels of 1-pentanol. This compound has a bitter almond flavor and a fatty taste, which can result in an unpleasant odor [38]. The UKW contained a high level of 3-methyl-1-butanol, which gives aromas of whisky, malt, and burnt notes [11]. Several C8 compounds, including 1-octen-3-one, 1-octen-3-ol, and 1-hydroxyoctan-3-one, impart a fresh mushroom off-flavor to wines [39]. The presence of linalool in UKW may impart a lavender and floral aroma to the fragrance [40]. The presence of D-citronellol in UKW results in a more intense rose flavor, compared to PKW [41]. The low odor threshold of rose oxide, more concentrated in UKW compared to PKW, results in a high ROAV, exerting a significant influence on the typical sweet rose flavor of the wine [40]. Some volatile phenols could impart negative notes to the wine aroma but could be beneficial anyway, in small concentrations. One example is given by the antioxidant 2,4-di-tert-butylphenol, a component characteristic of grape wines such as Cabernet Sauvignon, which could enhance the antimicrobial properties of the product [42].

Focusing on the taste of KW, E-tongue analysis was found to be nicely tailored in discriminating the two types of KW. UKW had higher sourness levels, while PKW showed more saltiness and bitterness, with potential repercussions for the perceived quality of the product. The principal source of sourness of wines is generally attributed to organic acids, some of which play a key role in determining fruit wines’ complexity and flavor balance [18]. They are primarily derived from lipolysis and proteolysis in the fruit and are the main precursors of numerous esters, aldehydes, and alcohols [42]. Previous studies have shown that tartaric acid is the main organic acid in kiwifruit [43] and, consequently, in KW [44]. Peel is particularly rich in tartaric acid, so a high extraction rate of peel could lead to elevated concentrations in the final product [28]. Depending on metal ions [45] and alcohol concentration, tartaric acid could give rise to insoluble salts, whose precipitation may have negative consequences on the perceived quality of the product [46]. Fumarate is a key intermediate of Krebs and urea cycles and can be absorbed by yeast and converted to malate. On the other hand, fumarate is structurally similar to malate, thus acting as a competitive inhibitor of the active site of malic enzyme lactase [47]. Galactarate is produced mainly from pectin (D-galacturonic acid) in the pericarp by the oxidation of galactose with nitric acid or from D-galacturonic acid by electrolytic oxidation. We suspect the abundance of pectin could result in a higher level of galactarate in UKW than PKW [48].

Amino acids are commonly present in fruit wines and play an important role in their taste [49]. Consistently with the present work, previous studies have detected aspartate, alanine, valine, tyrosine leucine, and isoleucine in KW [16,50]. Aspartate is the principal component responsible for the umami taste in KW, while alanine mainly contributes to sweetness, and the branched amino acids valine, leucine, and isoleucine impact bitterness [51]. In addition, leucine could be catabolized to *α*-keto acids, with a cheese-like odor, in turn, metabolized to aldehydes, alcohols, and carboxylic acids [38]. Leucine is also involved in the formation of isoamyl alcohol and its isomer, amyl alcohol [50].

## 5. Conclusions

The study is the first to employ multiple techniques in parallel to investigate the effect of kiwifruit peel on the flavor of KW. The peel was found to impart beneficial scents to the KW, such as giving it a stronger rose aroma. Overall, the E-tongue showed that the taste of the two types of KW can be effectively distinguished. The two types of KW differed in non-volatile organic compounds. In comparison to PKW, UKW presented lower levels of oxipurinol but higher levels of tartrate, galactarate, N-acetylserotonin, 4-hydroxy-3-methoxymandelate, fumarate, and N-acetylglycine. Despite several advantages, KW fermented with peel may affect the clarification of KW due to its abundant pectin content. Therefore, further studies would benefit from combining multiple techniques, to provide a comprehensive assessment of the quality of KW. Finally, this study could shed light on the utilization of waste in the kiwi deep-processing industry.

## Figures and Tables

**Table 1:** Volatile molecules with a significantly different concentration among the shared molecules by UKW and PKW.

Compound Name	Relative Concentration		*p*-Value
	PKW	UKW	
Ethyl heptanoate	1.23 × 10^−3^ ± 2.28 × 10^−4^	1.99 × 10^−3^ ± 3.52 × 10^−4^	0.005
Isoamyl acetate	5.09 × 10^−2^ ± 8.79 × 10^−3^	4.07 × 10^−2^ ± 5.60 × 10^−3^	0.002
Hexyl acetate	3.84 × 10^−3^ ± 7.99 × 10^−4^	2.40 × 10^−3^ ± 7.42 × 10^−4^	0.005
D-Citronellol	4.31 × 10^−4^ ± 1.78 × 10^−4^	1.37 × 10^−3^ ± 2.69 × 10^−4^	0.000
2,4,5-Trichlorotoluene	9.08 × 10^−4^ ± 2.10 × 10^−4^	6.02 × 10^−4^ ± 9.21 × 10^−5^	0.002
Rose oxide	2.68 × 10^−4^ ± 6.75 × 10^−5^	8.51 × 10^−4^ ± 2.64 × 10^−4^	0.000
Bornylene	3.59 × 10^−4^ ± 5.73 × 10^−5^	6.11 × 10^−4^ ± 1.10 × 10^−4^	0.002

**Table 2:** Concentration (mmol/L) of the molecules characterized by ^1^H-NMR with significant differences between UKW and PKW.

Compound Name	Molecular Concentration		*p*-Value
	PKW	UKW	
Oxypurinol	4.39 × 10^−2^ ± 1.16 × 10^−2^	2.93 × 10^−2^ ± 6.52 × 10^−3^	0.035
Fumarate	1.24 × 10^−2^ ± 1.53 × 10^−3^	1.68 × 10^−2^ ± 2.20 × 10^−3^	0.010
Tartrate	3.49 × 10^−2^ ± 5.32 × 10^−3^	4.40 × 10^−2^ ± 5.63 × 10^−3^	0.035
N-Acetylserotonin	2.71 × 10^−2^ ± 6.00 × 10^−3^	3.97 × 10^−2^ ± 9.00 × 10^−3^	0.031
4-Hydroxy-3-methoxymandelate	5.65 × 10^−2^ ± 7.17 × 10^−3^	7.98 × 10^−2^ ± 1.10 × 10^−2^	0.005
N-Acetylglycine	1.70 ± 9.69 × 10^−2^	2.17 ± 0.15	0.001
Galactarate	0.17 ± 6.93 × 10^−3^	0.22 ± 5.05 × 10^−2^	0.009

## Data Availability

The original contributions presented in the study are included in the article/Supplementary Material, further inquiries can be directed to the corresponding authors.

## Supplementary Materials

The following supporting information can be downloaded at: https://www.mdpi.com/article/10.3390/foods13111729/s1, Table S1. Relative content based on GC-MS characterization (Mean ± SD); Table S2. ROAV of VOCs in KW with/without peel; Table S3. Molecular concentrations based on ^1^H-NMR characterization (Mean ± SD).
