# Supplementary material for: Comparison of Aroma and Taste Profiles of Kiwi Wine Fermented with/without Peel by Combining Intelligent Sensory, Gas Chromatography-Mass Spectrometry, and Proton Nuclear Magnetic Resonance
Source: Foods. 2024 May 31;13(11):1729. doi: 10.3390/foods13111729 (PMC11172059; doi:10.3390/foods13111729)
Supplement: Supplementary file 1 [file foods-13-01729-s001.zip › foods-3022260-supplementary.pdf]

Comparison of Aroma and Taste Profiles of Kiwi Wine Fermented with/without Peel by Combining Intelligent Sensory, Gas Chromatography-Mass Spectrometry, and Proton Nuclear Magnetic Resonance

Bingde Zhou [1], Xiaochen Liu [1], Qiuyu Lan [1,2], Fang Wan [1], Zhibo Yang [1,3], Xin Nie [4], Zijian Cai [1], Bin Hu [3], Junni Tang [1], Chenglin Zhu [1,*] and Luca Laghi [2,*]

Supporting Materials

Table S1. Relative content based on GC-MS characterization (Mean ± SD).

Compound Name	Cas	RT	SI	RSI	Relative content	
					PKW	UKW
**Esters**						
Phenethyl acetate	103-45-7	31.77	787	908	$1.07\times10^{-3} \pm 1.62\times10^{-4}$	$9.24\times10^{-4}\pm3.10\times10^{-4}$
Ethyl 2-hydroxy-4-methyl valerate	10348-47-7	21.87	646	900	$5.13\times10^{-4} \pm 2.39\times10^{-4}$	ND
Ethyl propanoate	105-37-3	3.80	626	944	$5.41\times10^{-4} \pm 1.79\times10^{-4}$	$6.03\times10^{-4}\pm5.52\times10^{-5}$
Ethyl butanoate	105-54-4	5.24	665	919	$5.50\times10^{-3} \pm 8.26\times10^{-4}$	$5.81\times10^{-3}\pm1.08\times10^{-3}$
Isobutyl hexanoate	105-79-3	13.74	819	901	$5.81\times10^{-4} \pm 1.10\times10^{-4}$	$5.60\times10^{-4}\pm1.14\times10^{-4}$
Ethyl heptanoate	106-30-9	13.23	878	917	$1.23\times10^{-3} \pm 2.28\times10^{-4}$	$1.99\times10^{-3}\pm3.52\times10^{-4}$
Ethyl caprylate	106-32-1	16.53	920	920	$0.49 \pm 6.04\times10^{-2}$	$0.50\pm3.97\times10^{-2}$
Ethyl laurate	106-33-2	32.16	884	885	$1.73\times10^{-3} \pm 2.99\times10^{-4}$	$5.56\times10^{-3}\pm4.38\times10^{-3}$
Methyl hexoate	106-70-7	9.07	827	861	$1.62\times10^{-3} \pm 3.09\times10^{-4}$	$2.15\times10^{-3}\pm3.79\times10^{-4}$

Ethyl isovalerate	108-64-5	5.98	760	878	$2.65×10^{-4} ± 9.10×10^{-5}$	$2.79×10^{-4}±5.41×10^{-5}$
Isobutyl acetate	110-19-0	4.73	894	923	$8.57×10^{-4} ± 8.58×10^{-5}$	$8.79×10^{-4}±1.20×10^{-4}$
Ethyl caprate	110-38-3	28.01	932	932	$7.50×10^{-2} ± 1.14×10^{-2}$	$8.56×10^{-2}±2.82×10^{-2}$
Methyl Caprate	110-42-9	25.46	861	908	$6.46×10^{-4} ± 1.68×10^{-4}$	$9.27×10^{-4}±1.60×10^{-4}$
Methyl octylate	111-11-5	14.79	898	921	$6.71×10^{-3} ± 2.34×10^{-3}$	$1.14×10^{-2}±9.22×10^{-4}$
Diethyl succinate	123-25-1	29.28	867	885	$7.42×10^{-4} ± 2.49×10^{-4}$	$6.91×10^{-4}±1.73×10^{-4}$
Ethyl nonanoate	123-29-5	21.25	897	908	$2.02×10^{-3} ± 3.92×10^{-4}$	$3.48×10^{-3}±1.97×10^{-3}$
Ethyl hexanoate	123-66-0	10.45	924	924	$0.11 ± 1.33×10^{-2}$	$0.13±2.71×10^{-2}$
Isoamyl acetate	123-92-2	7.27	926	926	$5.09×10^{-2} ± 8.79×10^{-3}$	$4.07×10^{-2}±5.60×10^{-3}$
Ethyl tetradecanoate	124-06-1	34.44	770	844	$3.31×10^{-4} ± 1.16×10^{-4}$	$7.57×10^{-4}±5.75×10^{-4}$
Ethyl Acetate	141-78-6	2.84	891	966	$1.20×10^{-2} ± 2.00×10^{-3}$	$1.22×10^{-2}±2.62×10^{-3}$
Hexyl acetate	142-92-7	11.56	933	938	$3.84×10^{-3} ± 7.99×10^{-4}$	$2.40×10^{-3}±7.42×10^{-4}$
Isopentyl octanoate	2035-99-6	28.61	895	898	$2.83×10^{-3} ± 7.06×10^{-4}$	$4.62×10^{-3}±8.77×10^{-4}$
Isopentyl hexanoate	2198-61-0	17.29	869	883	$1.64×10^{-3} ± 1.47×10^{-4}$	$1.98×10^{-3}±5.70×10^{-4}$
Isoamyl decanoate	2306-91-4	32.40	913	920	$6.11×10^{-4} ± 2.34×10^{-4}$	$1.06×10^{-3}±2.81×10^{-4}$
Isobutyl octanoate	5461-06-3	22.29	765	834	$1.29×10^{-3} ± 1.90×10^{-4}$	$1.07×10^{-3}±2.08×10^{-4}$
Ethyl palmitate	628-97-7	36.22	770	802	$4.10×10^{-4} ± 6.65×10^{-5}$	$1.55×10^{-3}±9.76×10^{-4}$
Ethyl 9-decenoate	67233-91-4	29.50	843	935	$3.78×10^{-3} ± 2.31×10^{-3}$	ND
Propanoic acid, 2-methyl-, 1-(1,1-dimethylethyl)-2-methyl-1,3-propanediyl ester	74381-40-1	32.57	848	923	ND	$3.65×10^{-4}±9.75×10^{-5}$
Ethyl (E)-4-decenoate	76649-16-6	29.47	840	841	ND	$2.46×10^{-3}±1.31×10^{-3}$
Hex-2-enoic acid ethyl ester	1552-67-6	13.55	826	864	$4.48×10^{-4} ± 9.63×10^{-5}$	$7.22×10^{-4}±4.26×10^{-4}$
Ethyl 2-methylpropanoate	97-62-1	3.87	857	905	$3.95×10^{-4} ± 2.05×10^{-4}$	$2.69×10^{-4}±5.00×10^{-5}$
**Alcohols**						
2-Ethylhexanol	104-76-7	18.87	887	915	$6.16×10^{-4} ± 5.70×10^{-5}$	$7.06×10^{-4}±1.65×10^{-4}$

1-Hexanol	111-27-3	13.88	939	943	$1.87 \times 10^{-2} \pm 3.06 \times 10^{-3}$	$2.89 \times 10^{-2} \pm 1.20 \times 10^{-2}$
D-Citronellol	1117-61-9	31.07	728	872	$4.31 \times 10^{-4} \pm 1.78 \times 10^{-4}$	$1.37 \times 10^{-3} \pm 2.69 \times 10^{-4}$
1-Decanol	112-30-1	30.97	780	853	ND	$2.72 \times 10^{-4} \pm 5.87 \times 10^{-5}$
3-Methyl-1-butanol	123-51-3	10.00	915	915	ND	$9.56 \times 10^{-2} \pm 1.48 \times 10^{-2}$
1-Nonanol	143-08-8	28.81	723	856	$2.48 \times 10^{-4} \pm 3.57 \times 10^{-5}$	$9.63 \times 10^{-4} \pm 7.34 \times 10^{-4}$
5-Methyl-3-heptanol	18720-65-5	15.04	773	933	ND	$4.37 \times 10^{-3} \pm 3.11 \times 10^{-3}$
1-Octen-3-ol	3391-86-4	17.08	688	839	ND	$4.77 \times 10^{-4} \pm 1.12 \times 10^{-4}$
methionol	505-10-2	30.10	768	833	$5.93 \times 10^{-4} \pm 6.04 \times 10^{-4}$	$5.37 \times 10^{-4} \pm 4.65 \times 10^{-4}$
3-Hexen-1-ol	544-12-7	14.16	868	931	$7.47 \times 10^{-4} \pm 1.46 \times 10^{-4}$	$1.30 \times 10^{-3} \pm 4.75 \times 10^{-4}$
Phenethyl alcohol	60-12-8	33.06	954	963	$2.16 \times 10^{-2} \pm 1.58 \times 10^{-2}$	$2.12 \times 10^{-2} \pm 9.54 \times 10^{-3}$
1-Pentanol	71-41-0	9.93	911	927	$0.11 \pm 2.44 \times 10^{-2}$	ND
Linalool	78-70-6	22.30	843	865	ND	$1.63 \times 10^{-3} \pm 6.83 \times 10^{-4}$
trans-2-Hexenol	928-95-0	15.48	719	919	ND	$4.37 \times 10^{-4} \pm 1.73 \times 10^{-4}$
**Ketones**						
Cyclohexanone	108-94-1	11.93	873	911	$9.06 \times 10^{-4} \pm 2.65 \times 10^{-4}$	ND
2-Hydroxy-5-methylacetophenone	1450-72-2	35.89	701	870	$2.82 \times 10^{-4} \pm 3.24 \times 10^{-5}$	ND
1-(4-Hydroxy-3-methylphenyl) ethanone	876-02-8	35.88	653	779	ND	$3.29 \times 10^{-4} \pm 3.03 \times 10^{-5}$
3-Pentanone	96-22-0	4.12	820	867	$3.03 \times 10^{-4} \pm 8.33 \times 10^{-5}$	$3.59 \times 10^{-4} \pm 1.34 \times 10^{-4}$
**Aldehydes**						
Decanal	112-31-2	19.22	826	890	$1.63 \times 10^{-3} \pm 5.46 \times 10^{-4}$	$2.12 \times 10^{-3} \pm 9.79 \times 10^{-4}$
Undecanal	112-44-7	26.22	629	801	$3.89 \times 10^{-4} \pm 1.23 \times 10^{-4}$	ND
Dodecanal	112-54-9	29.90	885	939	$8.89 \times 10^{-4} \pm 6.58 \times 10^{-4}$	$6.44 \times 10^{-4} \pm 3.62 \times 10^{-4}$
Octanal	124-13-0	12.07	866	950	$1.73 \times 10^{-3} \pm 3.52 \times 10^{-4}$	ND
Nonanal	124-19-6	15.01	931	936	$1.59 \times 10^{-2} \pm 4.84 \times 10^{-3}$	$1.18 \times 10^{-2} \pm 9.43 \times 10^{-3}$

2-Undecenal	2463-77-6	30.66	872	910	$1.22×10^{-3}$ ± $7.62×10^{-4}$	ND
2,5-Dimethylbenzaldehyde	5779-94-2	31.67	828	922	ND	$7.91×10^{-4}$±$2.40×10^{-4}$
Hexanal	66-25-1	6.29	772	841	$4.87×10^{-4}$ ± $1.04×10^{-4}$	ND
**Acids**						
Nonanoic acid	112-05-0	35.53	913	915	$1.11×10^{-2}$ ± $1.31×10^{-2}$	$5.77×10^{-3}$±$6.91×10^{-3}$
Octanoic acid	124-07-2	34.58	918	923	$4.00×10^{-3}$ ± $1.76×10^{-3}$	$3.45×10^{-3}$±$1.66×10^{-3}$
Hexanoic acid	142-62-1	32.25	899	924	$2.18×10^{-3}$ ± $7.99×10^{-4}$	$2.75×10^{-3}$±$2.40×10^{-4}$
Lauric acid	143-07-7	38.72	689	828	$3.38×10^{-4}$ ± $8.23×10^{-5}$	ND
Decanoic acid	334-48-5	36.42	908	936	$3.02×10^{-3}$ ± $2.27×10^{-3}$	$2.36×10^{-3}$±$2.44×10^{-3}$
Ethylboronic acid	4433-63-0	6.88	800	822	$2.86×10^{-3}$ ± $4.56×10^{-4}$	$2.41×10^{-3}$±$4.57×10^{-4}$
2-Methylhexanoic acid	4536-23-6	29.09	684	748	$6.18×10^{-4}$ ± $3.65×10^{-4}$	$3.13×10^{-4}$±$6.02×10^{-5}$
**Others**						
Propylbenzene	103-65-1	9.49	873	913	$4.33×10^{-4}$ ± $7.38×10^{-5}$	$4.85×10^{-4}$±$1.55×10^{-4}$
p-Xylene	106-42-3	8.83	892	921	$4.21×10^{-4}$ ± $6.79×10^{-5}$	$6.16×10^{-4}$±$1.25×10^{-4}$
4-Ethyltoluene	622-96-8	10.96	888	909	ND	$8.10×10^{-4}$±$4.97×10^{-4}$
2,4,5-Trichlorotoluene	6639-30-1	30.73	847	905	$9.08×10^{-4}$ ± $2.10×10^{-4}$	$6.02×10^{-4}$±$9.21×10^{-5}$
2,3,4-Trichlorotoluene	7359-72-0	32.05	867	899	$7.20×10^{-4}$ ± $1.09×10^{-4}$	$8.41×10^{-4}$±$1.80×10^{-4}$
1,2,4-Trimethylbenzene	95-63-6	11.02	783	798	$1.28×10^{-3}$ ± $1.64×10^{-4}$	$2.19×10^{-3}$±$1.21×10^{-3}$
Hemimellitene	526-73-8	10.89	750	841	ND	$1.00×10^{-3}$±$7.51×10^{-4}$
Pentylcyclopropan	2511-91-3	22.96	840	902	$8.78×10^{-4}$ ± $8.17×10^{-5}$	$9.98×10^{-4}$±$2.68×10^{-4}$
1,1,3,3,5,5,7,7,9,9,11,11,13,13,15,15-Hexadecamethyloctasiloxane	19095-24-0	34.15	736	794	$5.80×10^{-4}$ ± $1.00×10^{-4}$	$7.46×10^{-4}$±$1.32×10^{-4}$
Tetradecamethyl Cycloheptasiloxane	107-50-6	25.90	771	792	$1.68×10^{-3}$ ± $3.04×10^{-4}$	$2.32×10^{-3}$±$7.47×10^{-4}$

Dodecamethylcyclohexasiloxane	540-97-6	14.83	736	787	6.42×10^{-3} ± 3.80×10^{-3}	1.24×10^{-2}±6.28×10^{-3}
Decamethylcyclopentasiloxane	541-02-6	9.83	842	916	ND	2.89×10^{-3}±1.71×10^{-3}
Hexamethylcyclotrisiloxane	541-05-9	2.56	941	950	4.22×10^{-3} ± 1.21×10^{-3}	4.48×10^{-3}±1.14×10^{-3}
Octamethylcyclotetrasiloxane	556-67-2	5.06	941	962	3.96×10^{-3} ± 6.51×10^{-4}	4.65×10^{-3}±2.17×10^{-3}
Hexadecamethylcyclooctasiloxane	556-68-3	30.83	838	870	1.92×10^{-3} ± 3.42×10^{-4}	2.23×10^{-3}±4.06×10^{-4}
Octadeamethyl-cyclononasiloxane	556-71-8	32.73	853	869	1.26×10^{-3} ± 2.43×10^{-4}	1.24×10^{-3}±4.10×10^{-4}
2,4-Di-tert-butylphenol	96-76-4	36.77	926	934	4.26×10^{-3} ± 1.18×10^{-3}	4.27×10^{-3}±1.19×10^{-3}
2,4-Di-tert-butyl-6-nitrophenol	20039-94-5	34.84	763	867	3.76×10^{-4} ± 1.41×10^{-4}	ND
Rose oxide	16409-43-1	13.64	696	835	2.68×10^{-4} ± 6.75×10^{-5}	8.51×10^{-4}±2.64×10^{-4}
2-Chlorohydrocinnamonitrile	7315-17-5	40.32	620	813	7.49×10^{-4} ± 7.60×10^{-5}	9.11×10^{-4}±2.14×10^{-4}
Bornylene	464-17-5	19.88	696	851	3.59×10^{-4} ± 5.73×10^{-5}	6.11×10^{-4}±1.10×10^{-4}
Coumaran	496-16-2	37.76	798	854	5.05×10^{-4} ± 2.02×10^{-4}	5.43×10^{-4}±1.74×10^{-4}

"ND" indicates that the substance was not detected.

The relative odor activity values (ROAV) of VOCs in KW were calculated using the following formula [1].

$$ROAV = 100 \times \frac{C_A}{C_{stan}} \times \frac{T_{stan}}{T_A}$$

C_A: The relative content of VOCs in the KW sample.
C_{stan}: The relative content of VOCs that contribute most to the overall flavor.
T_{stan}: The odor threshold of VOCs that contribute most to the overall flavor.
T_A: The odor threshold of VOCs in the KW sample.

Table S2. ROAV of VOCs in KW with/without peel.

Compound Name	Cas	Descriptor	Aroma threshold (μg/L)	ROAV	
				PKW	UKW
Phenethyl acetate	103-45-7	rose	250	0.225317	0.178642
Ethyl 2-hydroxy-4-methyl valerate	10348-47-7	blackberry	10.12	2.530065	-
Ethyl butanoate	105-54-4	sweet	82	3.434718	3.50991
Ethyl heptanoate	106-30-9	pineapple	13153	0.004672	0.007545
Ethyl caprylate	106-32-1	apricot, banana, pear	250	100	100
Ethyl laurate	106-33-2	fruity, floral	400	0.217636	0.69472
Ethyl isovalerate	108-64-5	fruity	7	2.194651	2.126694
Isobutyl acetate	110-19-0	apple, banana, glue, pear	2100	0.021947	0.021267
Diethyl succinate	123-25-1	wine, fruity	353193	0.000101	0.000098
Ethyl nonanoate	123-29-5	grape	3151	0.032503	0.055119
Ethyl hexanoate	123-66-0	apple, raw banana, pineapple, floral	210	27.09175	30.26995
Isoamyl acetate	123-92-2	banana	94	27.72887	21.48565
Ethyl tetradecanoate	124-06-1	sweet	180	0.085348	0.220546
Ethyl acetate	141-78-6	pineapple	32551	0.018878	0.018598
Hexyl acetate	142-92-7	apple, banana, grass, herb, pear	670	0.290436	0.177754

Ethyl palmitate	628-97-7	apple, sweet	1000	0.020483	0.074434
Ethyl 9-decenoate	67233-91-4	fruity	100	1.945924	-
1-Hexanol	111-27-3	banana, floral, grass	1300	0.736615	1.103154
1-Decanol	112-30-1	floral	400	-	0.037217
3-Methyl-1-butanol	123-51-3	whisky, malt, burnt	50000	-	0.094879
1-Nonanol	143-08-8	/	5000	0.002048	0.009925
1-Octen-3-ol	3391-86-4	fresh mushroom	6.12	-	4.054156
Phenethyl alcohol	60-12-8	rose	14000	0.079007	0.075143
1-Pentanol	71-41-0	bitter almond, fatty	80000	0.07118	-
Linalool	78-70-6	floral	5300	-	0.01498
Decanal	112-31-2	oil, soap, orange peel	1.25	65.54691	83.36642
Octanal	124-13-0	citrus, grassy	39	2.232166	-
Nonanal	124-19-6	melon	122	6.673897	4.799589
Octanoic acid	124-07-2	cheese, fatty	2701	0.075836	0.064302
Hexanoic acid	142-62-1	barbecue, cheese	8500	0.013254	0.015763
Lauric acid	143-07-7	metal	9154	0.001678	-
Decanoic acid	334-48-5	rancid, fatty	13737	0.011183	0.00867
2,4-Di-tert-butylphenol	96-76-4	/	200	1.100983	1.066892
Rose oxide	16409-43-1	rose	0.2	76.81278	223.3029

"-" indicates that the substance was not detected. "/" indicates that no information on the substance was sought.

Table S3. Molecular concentrations based on ^{1}H-NMR characterization (Mean ± SD).

Compound Name	Molecular concentration	
	PKW	UKW
**Amino acids, peptides, and analogs**		
Tryptophan	$6.05\times10^{-2} \pm 2.92\times10^{-2}$	$3.99\times10^{-2} \pm 2.45\times10^{-2}$
Phenylalanine	$0.23 \pm 6.77\times10^{-2}$	$0.17 \pm 6.29\times10^{-2}$
Tyrosine	$0.19 \pm 2.14\times10^{-2}$	$0.18 \pm 2.48\times10^{-2}$
Ornithine	$3.32\times10^{-2} \pm 9.86\times10^{-3}$	$3.26\times10^{-2} \pm 1.06\times10^{-2}$
Creatine	$4.38\times10^{-2} \pm 4.73\times10^{-3}$	$4.54\times10^{-2} \pm 6.94\times10^{-3}$
Creatinine	$0.17 \pm 1.25\times10^{-2}$	$0.16 \pm 4.28\times10^{-2}$
Aspartate	$0.19 \pm 1.84\times10^{-2}$	$0.18 \pm 2.90\times10^{-2}$
Alanine	1.01 ± 0.15	0.86 ± 0.19
Sarcosine	$4.03\times10^{-2} \pm 3.21\times10^{-3}$	$3.82\times10^{-2} \pm 5.67\times10^{-3}$
Valine	$0.18 \pm 3.63\times10^{-2}$	$0.13 \pm 3.62\times10^{-2}$
Isoleucine	$0.16 \pm 4.68\times10^{-2}$	$0.13 \pm 3.51\times10^{-2}$
Leucine	0.38 ± 0.12	0.28 ± 0.10
N, N-Dimethylglycine	$3.07\times10^{-2} \pm 1.02\times10^{-3}$	$3.28\times10^{-2} \pm 2.64\times10^{-3}$
N-Acetylglycine	$1.70\pm 9.69\times10^{-2}$	2.17 ± 0.15
**Carbohydrates and derivatives**		
Galactose	0.68 ± 0.32	0.39 ± 0.30
Glucose	$0.25 \pm 4.17\times10^{-2}$	0.28 ± 0.11
Lactose	$0.10 \pm 5.33\times10^{-2}$	$6.42\times10^{-2} \pm 3.64\times10^{-2}$
Xylose	1.25 ± 0.70	0.72 ± 0.61
Fucose	$0.25 \pm 6.00\times10^{-2}$	$0.20 \pm 3.75\times10^{-2}$
Arabinose	0.87 ± 0.12	0.70 ± 0.35
Lactulose	$0.29 \pm 3.49\times10^{-2}$	$0.26 \pm 5.07\times10^{-2}$

1,6-Anhydro-*β*-D-glucose	$1.34\times10^{-2} \pm 2.43\times10^{-3}$	$1.64\times10^{-2} \pm 9.13\times10^{-3}$
Fructose	1.88 ± 0.11	1.43 ± 0.42
**Organic acids and derivatives**		
Formate	$9.29\times10^{-2} \pm 9.07\times10^{-3}$	$0.10 \pm 2.58\times10^{-2}$
Acetate	4.87 ± 1.73	3.67 ± 1.18
Butyrate	$0.40 \pm 5.00\times10^{-2}$	$0.36 \pm 6.48\times10^{-2}$
Fumarate	$1.24\times10^{-2} \pm 1.53\times10^{-3}$	$1.68\times10^{-2} \pm 2.20\times10^{-3}$
Tartrate	$3.49\times10^{-2} \pm 5.32\times10^{-3}$	$4.40\times10^{-2} \pm 5.63\times10^{-3}$
Pantothenate	$0.40 \pm 9.79\times10^{-2}$	0.42 ± 0.22
Malonate	$2.39\times10^{-2} \pm 5.19\times10^{-3}$	$1.71\times10^{-2} \pm 4.65\times10^{-3}$
Malate	$1.28 \pm 8.92\times10^{-2}$	1.46 ± 0.40
Pyroglutamate	$5.89\times10^{-2} \pm 3.03\times10^{-2}$	$5.25\times10^{-2} \pm 1.51\times10^{-2}$
Succinate	5.36 ± 0.51	5.11 ± 0.20
Isocaproate	$1.36 \pm 9.19\times10^{-2}$	1.21 ± 0.22
2-Hydroxyisovalerate	$5.70\times10^{-2} \pm 9.18\times10^{-3}$	$4.23\times10^{-2} \pm 1.24\times10^{-2}$
4-Aminobutyrate	0.54 ± 0.13	0.51 ± 0.14
Lactate	1.05 ± 0.63	0.88 ± 0.61
Indole-3-acetate	$3.49\times10^{-2} \pm 1.77\times10^{-2}$	$3.67\times10^{-2} \pm 1.79\times10^{-2}$
4-Hydroxyphenyllactate	$0.27 \pm 1.39\times10^{-2}$	$0.26 \pm 1.42\times10^{-2}$
trans-Aconitate	$4.72\times10^{-2} \pm 3.61\times10^{-3}$	$4.59\times10^{-2} \pm 6.19\times10^{-3}$
Epicatechin	$1.99\times10^{-2} \pm 6.40\times10^{-3}$	$2.73\times10^{-2} \pm 6.75\times10^{-3}$
5-Aminolevulinate	$0.19 \pm 1.85\times10^{-2}$	$0.19 \pm 3.17\times10^{-2}$
Galactonate	1.03 ± 0.18	1.13 ± 0.27
Galactarate	$0.17 \pm 6.93\times10^{-3}$	$0.22 \pm 5.05\times10^{-2}$
2-Oxoglutarate	0.67 ± 0.12	$0.63 \pm 9.63\times10^{-2}$
2-Methylglutarate	$0.14 \pm 1.55\times10^{-2}$	$0.14 \pm 2.36\times10^{-2}$

Methionine	0.12 ± 2.25×10^{-2}	9.83×10^{-2} ± 3.00×10^{-2}
4-Hydroxy-3-methoxymandelate	5.65×10^{-2} ± 7.17×10^{-3}	7.98×10^{-2} ± 1.10×10^{-2}
Syringate	0.11 ± 1.81×10^{-2}	0.12 ± 1.21×10^{-2}
**Nucleosides, nucleotides, and analogs**		
Guanosine	2.03×10^{-2} ± 8.67×10^{-3}	2.43×10^{-2} ± 3.26×10^{-2}
Cytidine	3.01×10^{-2} ± 3.77×10^{-3}	2.41×10^{-2} ± 9.58×10^{-3}
Uridine	0.13 ± 2.01×10^{-2}	0.11 ± 4.32×10^{-2}
Uracil	8.93×10^{-2} ± 3.76×10^{-2}	7.66×10^{-2} ± 2.76×10^{-2}
Thymine	0.34 ± 2.86×10^{-2}	0.31 ± 4.38×10^{-2}
**Alcohols**		
Oxypurinol	4.39×10^{-2} ± 1.16×10^{-2}	2.93×10^{-2} ± 6.52×10^{-3}
Methanol	10.20 ± 0.69	12.00 ± 1.7
Ethanol	1166.00 ± 51.00	1177.49 ± 271.00
Propylene glycol	3.08 ± 0.24	3.05 ± 1.11
myo-Inositol	8.05 ± 0.31	7.81 ± 0.54
Glycerol	32.30 ± 2.05	33.10 ± 6.12
**Others**		
Hypoxanthine	3.58×10^{-2} ± 1.59×10^{-2}	2.70×10^{-2} ± 8.92×10^{-3}
Xanthine	5.26×10^{-2} ± 6.33×10^{-3}	4.23×10^{-2} ± 1.11×10^{-2}
N-Acetylserotonin	2.71×10^{-2} ± 6.00×10^{-3}	3.97×10^{-2} ± 9.00×10^{-3}
N-Acetylglucosamine	0.60 ± 8.25×10^{-2}	0.77 ± 0.14
1,3-Dihydroxyacetone	0.40 ± 5.38×10^{-2}	0.42 ± 0.20
Choline	0.45 ± 4.24×10^{-2}	0.42 ± 5.70×10^{-2}
Dimethyl sulfone	2.69×10^{-2} ± 1.62×10^{-3}	2.63×10^{-2} ± 2.76×10^{-3}
Ethanolamine	0.19 ± 1.50×10^{-2}	0.18 ± 1.01×10^{-2}
Asparagine	8.82×10^{-2} ± 1.36×10^{-2}	8.59×10^{-2} ± 2.25×10^{-2}

Methylamine	$1.50\times10^{-2} \pm 2.05\times10^{-3}$	$1.60\times10^{-2} \pm 1.01\times10^{-3}$
Pyridoxine	$2.10\times10^{-2} \pm 6.12\times10^{-3}$	$1.90\times10^{-2} \pm 5.16\times10^{-3}$
Acetone	$8.10\times10^{-2} \pm 9.22\times10^{-2}$	0.12 ± 0.11
Acetoin	0.11 ± 0.17	0.17 ± 0.18
Hydroxy acetone	$3.93\times10^{-2} \pm 3.65\times10^{-3}$	$3.92\times10^{-2} \pm 5.35\times10^{-3}$

Reference

1. Li, P.; Jia, Y.; Cai, D.; Wang, X.; Liu, J.; Zhu, R.; Wang, Z.; He, Y.; Wen, L. Study on the Relationship between Flavor Components and Quality of Ice Wine during Freezing and Brewing of “beibinghong” Grapes. *Food Chem X* **2023**, *20*, 101016, doi:10.1016/J.FOCHX.2023.101016.
